# Impact of real-world implementation of evidence-based insomnia treatment within a large payor-provider health system: initial provider and patient-level outcomes

**DOI:** 10.1093/sleepadvances/zpae053

**Published:** 2024-07-27

**Authors:** Bradley E Karlin, Ryan J Anderson, Jillian M Rung, Charlotte Drury-Gworek, Tyson S Barrett

**Affiliations:** Enterprise Behavioral Health, Highmark Health, Pittsburgh, PA, USA; Department of Mental Health, Bloomberg School of Public Health, Johns Hopkins University, Baltimore, MD, USA; Enterprise Behavioral Health, Highmark Health, Pittsburgh, PA, USA; Enterprise Data & Analytics, Highmark Health, Pittsburgh, PA, USA; Enterprise Data & Analytics, Highmark Health, Pittsburgh, PA, USA; Enterprise Data & Analytics, Highmark Health, Pittsburgh, PA, USA

## Abstract

**Study Objectives:**

Insomnia has substantial and wide-ranging negative effects on clinical and functional outcomes and on health care expenditures, yet few individuals receive gold-standard insomnia treatment. The current article examines provider and patient outcomes associated with real-world implementation of Cognitive Behavioral Therapy for Insomnia (CBT-I), as part of a pilot initiative designed to establish initial capability for evidence-based insomnia treatment within one of the largest payor-provider systems in the United States.

**Methods:**

Provider training outcomes were assessed using the CBT-I Competency Rating Scale and self-report measures. Patient outcomes were assessed using the Insomnia Severity Index (ISI) and Patient Health Questionnaire-9.

**Results:**

All clinicians (*N *= 11) achieved competency in CBT-I and reported large increases in knowledge and confidence related to insomnia and insomnia treatment. Clinicians also reported high intention to deliver CBT-I and significant improvements in overall job satisfaction following competency-based CBT-I training. Among all patients who initiated CBT-I (*N = *48), mixed effects modeling demonstrated significant reduction in average ISI scores (12.57 to 5.88, SEs = 1.08-1.14). More than two-thirds of patients (68.8%) completed treatment. Among completers of this brief treatment, mean insomnia severity improvement was 71% (Hedges *g* = 1.56).

**Conclusions:**

Findings provide support for the feasibility and effectiveness of real-world CBT-I implementation, extending past findings to a private, payor-provider context. Patient and provider-level outcomes suggest the significant opportunity private systems may have in increasing the availability of gold-standard treatment for insomnia.

Approximately 1 in 3 adults and 1 in 2 older adults experience symptoms of insomnia [1, 2]. As many as half of those with insomnia symptoms have conditions consistent with an insomnia disorder [3]. The most common sleep-related problem, insomnia has considerable and wide-reaching consequences and, if not effectively treated, tends to be highly enduring in nature [4–7]. The effects of insomnia implicate numerous clinical (cardiovascular [8, 9], cognitive [10–15], psychological [13–15]), functional [16, 17], and quality of life [16, 18, 19] domains, and is associated with increased risk of falls and overall mortality, most notably in older individuals [20].

Beyond its substantial clinical and functional effects, a growing body of research has shown insomnia to be associated with significantly higher total health care expenditures, as well as higher costs associated with reduced workplace functioning (e.g. increased absenteeism, reduced presenteeism) and quality of life [21–23]. Notably, findings from a recent examination of the financial impact of insomnia, which informed decisions and plans for the implementation initiative evaluated in the current study, found that median total health care costs among individuals with insomnia were 4–6 times greater than among those without insomnia, and mean total costs were 2–3 times greater [24].

Although insomnia is highly untreated and undertreated, Cognitive Behavioral Therapy for Insomnia (CBT-I) is a brief (4–6 session), highly efficacious treatment for insomnia, as demonstrated in dozens of randomized controlled trials (RCTs) [25, 26]. In fact, the effect sizes reported in RCTs of CBT-I render it among the most efficacious psychological treatments in existence. In light of these findings, CBT-I is recommended at the highest level and identified as a first-line treatment in clinical practice guidelines and consensus statements [27–29]. CBT-I is a particularly attractive option for older adults due to its favorable safety profile relative to hypnotic medications [30]. Hypnotic medication use has been found to be related to increased risk of falls and fractures [31, 32], cognitive impairment [33, 34], and mortality [35]. In addition, increasing evidence demonstrates CBT-I to yield a number of secondary benefits beyond insomnia, including decreased levels of fatigue [36, 37], improved daytime functioning [36, 38], reductions in depression and other behavioral health conditions [39, 40], and increased quality of life [37, 41]. Beyond its clinical effects, CBT-I has also been shown to be associated with significant reductions in health care utilization and costs, thus representing important opportunities for cost savings [42, 43].

Despite its clinical and cost-effectiveness and broad utility, CBT-I remains widely unavailable on the clinical frontlines. Previously, we implemented the world’s largest training and delivery system of CBT-I within the Department of Veterans Affairs (VA) health care system, as part of a broader effort to transform the VA mental health care system into an evidence-based and recovery-oriented system of care [44]. This structured implementation of CBT-I was associated with robust improvements in provider competency to deliver the treatment, as well as large reductions in insomnia severity and improvements in depression, suicidal ideation, and quality of life among Veterans following this brief intervention [40, 45–47].

On the heels of the national implementation of CBT-I within the VA health care system, Highmark Health, the nation’s third-largest payor-provider health system, has developed a similar training and implementation initiative to improve psychological and physical health outcomes. This pilot initiative was designed to establish new capability for delivering evidence-based treatment for insomnia within the Allegheny Health Network (AHN), the provider system of Highmark Health. Findings from this implementation and new capability will be used to inform potential broader implementation of CBT-I. The initiative is part of an enterprise-wide Behavioral Health Strategy designed to transform and innovate the model of behavioral health by making behavioral health care more personalized, proactive, evidence-based, data-driven, and upstream-focused [48].

The overall goals of the current manuscript are to assess initial provider and patient outcomes associated with the real-world implementation of CBT-I within AHN and to assess the extent to which findings from the VA system may be replicated in a private health system context. It was anticipated that training in and implementation of CBT-I by newly trained clinicians would be associated with robust provider and patient outcomes, including significant improvements in provider preparedness to deliver CBT-I and robust improvements in insomnia and cooccurring depression among patients. Specifically, it was hypothesized that provider and patient outcomes from the current initiative would be comparable to the CBT-I training and implementation outcomes documented in the VA system, on which the current training and implementation approach was modeled.

## Method

### CBT-I protocol description

CBT-I is a structured, brief psychotherapy that targets unhelpful behaviors, thoughts, and emotions that disrupt the ability to initiate and maintain sleep. The treatment protocol used in the present study is based on a case formulation approach to insomnia treatment, including descriptive case examples, sample dialogue, and numerous therapist and patient resources to facilitate assessment, case conceptualization, treatment planning, treatment delivery, and progress monitoring [49]. The case formulation approach is distinguished by its focus on individualizing treatment to specific patient characteristics and needs, including considerations for treatment of patients with cooccurring conditions such as depression or anxiety and the use of cognitive components throughout treatment to address unhelpful thoughts and beliefs that contribute to hyperarousal, interfere with sleep physiology, and adversely affect adherence to core treatment components. This emphasis on fidelity with individualization to specific patient needs and preferences is designed to prevent rote, manualized application of technical skills and is consistent with prior successes in implementing evidence-based psychological treatments in real-world settings [45, 50].

The first session (session 0) consists of an intake session, which includes assessment of sleep history and screening for other sleep disorders. Findings from the initial evaluation are used to develop an individualized case conceptualization to guide treatment. Session 1 consists of the first treatment session.

### CBT-I training program description

The competency-based training model for the initiative was comprised of two phases, consistent with other large-scale competency-based EBP training models [44]. The first phase consisted of 3-day foundational workshop training that included didactic case-based instruction and experiential components. The second phase (training consultation) followed immediately after the foundational workshop training and consisted of five months of weekly, 90-minute case-based consultation sessions conducted in small groups of four therapists and led by expert training consultants from Stanford University who provided supportive and constructive feedback to therapists on their use of CBT-I skills and competencies, facilitated case-based discussion, and reinforced core skills through role play and assigned readings to facilitate skill acquisition and enrich the learning experience.

Therapists were required to enroll at least two training cases during consultation and capture audio-video recordings of all therapy sessions after obtaining patients’ written informed consent. Therapists enrolled patients 18 years of age and older with a primary presenting problem of insomnia and were encouraged to enroll patients with cooccurring anxiety and depression. Other cooccurring behavioral health conditions (e.g. posttraumatic stress disorder [PTSD], bipolar disorder) were allowable if the other condition was stable and, in the case of PTSD, the patient was not concurrently receiving prolonged exposure. Patients with seizure disorders, untreated substance use disorders, untreated severe obstructive sleep apnea, and medically unstable patients were not eligible to receive CBT-I. Both AHN patients who were Highmark members, as well as non-highmark members, were eligible to receive CBT-I.

During the training consultation phase, training consultants regularly reviewed and rated full-length, recorded therapy sessions using the 15-item CBT-I Competency Rating Scale (CBT-I-CRS) [45]. Training consultants reviewed up to seven full session recordings per therapist and made ratings for at least one assessment session and two therapy sessions or until the competency criteria were met within the training consultation period. Therapists were required to attend at least 75% of weekly consultation calls, enroll at least two training cases, complete at least six sessions with one case (or fewer if remission occurred prior to six sessions), submit at least six recorded sessions to consultants for review, meet the minimum threshold for core CBT-I competencies assessed by five items on the CBT-I-CRS, and obtain a CBT-I-CRS total score of 30 or higher. Successful completion of these requirements constituted full competency in treatment delivery and successful completion of the training program, following which therapists progressed to use of CBT-I as part of routine clinical practice.

Participation in the CBT-I Training and Implementation Initiative was voluntary and offered to master’s level licensed professional counselors, licensed clinical social workers, and licensed psychologists identified within integrated primary care and outpatient specialty behavioral health settings within AHN. Clinicians were provided with the opportunity to apply to participate in the training and implementation initiative. Clinicians were selected to participate based on their degree of interest in receiving competency-based training in CBT-I, general interest in CBT or other evidence-based psychotherapies (EBPs), and their willingness to at least minimally incorporate CBT-I into their current practice. Clinicians were not required to have received any formal EBP training or to meet a certain threshold for clinical experience or expertise. While participation was voluntary and did not include requirements for formal training or experience in EBPs, there was interest in recruiting interested clinicians to increase the likelihood of completion of the competency-based training process and of establishing a foundation of support and early adopters for ongoing treatment delivery and implementation. As a prerequisite to enrolling in the training program, providers were informed about the training process and asked to agree to training requirements. Given the nature of this as a pilot training and implementation initiative designed to develop and evaluate the net new capability to provide evidence-based treatment for insomnia, participation was not open to all clinicians interested in training but primarily focused on individuals willing to commit to ongoing delivery of CBT-I following training.

A total of 45 behavioral health therapists across primary care and specialty behavioral health settings were invited to apply to the program. In primary care, there were 35 therapists, all of whom were invited to apply. Of those, 8 applied and were accepted. The primary reason for not applying was concern about the time required for training and consultation. In outpatient specialty behavioral health settings, 10 therapists with established professional experience (≥3 years post-licensure) and demonstrated record of providing high-quality treatment for ≥12 months at AHN were invited to apply. Of the 10 therapists approached, 7 applied to the program. An eighth therapist expressed interest but was planning to retire and chose not to apply. Of the 7 therapists that applied, 4 were accepted into the training program and 3 were rejected due to timing and new role transitioning, lack of role fit with clinical focus area and unrelated work demands, and inability to commit to ongoing use of CBT-I following training (interest in training but not necessarily ongoing implementation).

A total of 12 clinicians were enrolled in the training program. One clinician left her position early in training to take a new role outside of AHN. Of the remaining 11 clinicians, all were female, three were licensed psychologists, one was a licensed clinical social worker, and seven were licensed professional counselors.

### Clinician-level measures

#### CBT-I competency.

The CBT-I CRS is a 15-item observer-rated measure of general and specific CBT-I competencies [45]. Training consultants completed the CBT-I CRS based on a review of video recordings of up to seven full sessions per therapist during the 5-month training consultation period. Five of the 15 CBT-I CRS items assess core CBT-I competencies: review of sleep diary data, presentation of stimulus control, presentation of time in bed restriction, implementation of cognitive therapy, and case conceptualization grounded in knowledge of sleep physiology. Examples of competencies assessed by other items include attention to adherence issues, interpersonal effectiveness of the therapist, and therapist collaboration with the patient. Items are rated on a five-point scale: 0 (“Poor”) to 4 (“Excellent”). The CBT-I CRS was developed as part of an expert consensus process for use in the national implementation of CBT-I within the VA health care system; consistent with prior use, a total score of 30 or higher *and* scores of 2 or higher on the five core items was used as the threshold for competency [45].

#### Knowledge, attitudes, treatment self-efficacy.

CBT-I knowledge, attitudes, and confidence to deliver CBT-I were assessed using an 18-item self-report measure adapted from prior competency-based evidence-based psychotherapy training programs [50, 51]. The measure was administered to therapists at three-time points: at the start of the foundational training workshop, at the conclusion of the foundational training workshop, and at the conclusion of the training consultation phase. Knowledge of insomnia and CBT-I and insomnia was evaluated with six items (e.g. “How knowledgeable are you about insomnia”) rated on a Likert scale, ranging from 1 (“Not at all knowledgeable”) to 7 (“Extremely knowledgeable”). Attitudes toward CBT-I were evaluated with four items (e.g. “CBT-I is an effective treatment for patients with insomnia”) rated on a Likert scale, ranging from 1 (“Strongly disagree”) to 7 (“Strongly agree”). Self-efficacy or confidence in delivering CBT-I was evaluated by eight items emphasizing core treatment competencies such as case conceptualization and implementation of stimulus control and time in bed restriction, with items rated on a Likert scale, ranging from 1 (“Not at all confident”) to 7 (“Extremely confident”).

### Patient-level measures

#### Patient demographics and health status.

Information related to patient demographics (age, sex, and race), ongoing/active behavioral and physical health diagnoses noted in the problem list, and insomnia-related medications were extracted from electronic health records. Behavioral health diagnoses included depressive and anxiety disorders, PTSD, alcohol use disorder, and non-alcohol substance use disorders, as well as non-insomnia sleep disorders. For physical health diagnoses, patients’ problem lists were reviewed for conditions included in the Charlson Comorbidity Index (CCI) [52], which includes 17 physical health conditions predictive of mortality (e.g. renal disease, metastatic cancers). This information was then used to calculate patients’ CCI scores, which is a sum of indicators based on the presence/absence of the physical health conditions, with individual conditions weighted by their severity (e.g. renal disease has a weighted value of 1, and metastatic cancers have a value of 6). Higher scores correspond to higher physical health disease impact. Lastly, whether a patient had an active insomnia-related prescription medication was also recorded. Insomnia-related medications included benzodiazepines FDA-approved for treatment of insomnia, nonbenzodiazepine sedative-hypnotics (i.e. Z-drugs), orexin receptor antagonists, melatonin receptor agonists, select antidepressants prescribed at a low dose for sleep (e.g. doxepin, trazodone), and select first-generation antihistamines commonly used to treat insomnia (e.g. doxylamine succinate).

#### Insomnia severity.

The Insomnia Severity Index (ISI) is a valid and reliable seven-item measure of insomnia severity [53]. ISI scores range from 0 to 28, with higher scores reflecting greater insomnia severity. The recommended cutoff showing the optimal classification of insomnia in clinical samples is 11 [53]. Moderate to marked improvement in insomnia is reflected by a decrease of at least 8 points in insomnia severity [54]. Therapists administered the ISI at the beginning of each session.

#### Depression severity.

The nine-item Patient Health Questionnaire (PHQ-9) is a measure of patient-reported symptoms of depression. Patients rate the frequency of depression symptoms on a four-point scale, ranging from 0 (“Not at all”) to 3 (“Nearly every day”). PHQ-9 scores range from 0 to 27, with higher scores reflecting higher depression severity. Scores of 0 to 4 are classified as “none to minimal,” 5 to 9 are classified as “mild,” 10 to 14 are classified as “moderate,” 15 to 19 are classified as “moderately severe,” and 20 to 27 are classified as “severe.” The PHQ-9 is a widely used, internally consistent, valid, and reliable measure of depression severity [54]. Therapists administered the PHQ-9 prior to the start of the intake session and prior to the start of the final session.

### Training program evaluation

Immediately following the training consultation process, therapists responded to seven self-report items assessing their satisfaction with the training program and their perceptions of the impact of the program on areas such as enhancement of their overall therapy skills, job satisfaction, and intent to incorporate CBT-I into ongoing practice. Items were rated on a Likert scale where a rating of 1 corresponded to the lowest possible degree of satisfaction and a rating of 7 corresponded to the highest possible degree of satisfaction.

### Evaluation procedures

Program evaluation staff assigned each therapist a unique training program identification number, and each patient was assigned a unique patient identification number. Training consultants reviewed therapists’ recorded sessions a minimum of four times for every therapist to determine competency to deliver core components of CBT-I. Program evaluation staff administered the training program evaluation measures to therapists at three separate time points. Responses were kept confidential, and de-identified data were analyzed and reported in aggregate.

Therapists informed program evaluation staff each time they enrolled a new patient and provided status updates at weekly intervals as patients progressed through treatment. Therapists obtained written informed consent from patients for audio-video recording of CBT-I sessions and release of information for training and consultation purposes. Patient responses to assessment measures (i.e. ISI, PHQ-9) were collected at the start of sessions and documented in the electronic medical record at AHN per standard clinical practice.

Clinical evaluation data, patient demographics, and medical history information were collected retrospectively via a one-time review of information already present in the administrative and clinical records at AHN. The Institutional Review Board at AHN determined that this research qualified for exempt status.

### Statistical analyses

Descriptive and inferential analyses were conducted using RStudio Workbench (v 1.4.1717-3) running *R* (v. 4.0), with select descriptive statistics calculated using Microsoft Excel (v 2301). An alpha of.05 was used for all inferential analyses; *p*-values for follow-up *t-*tests were Bonferroni-corrected. For all inferential analyses, assumptions were verified by performing the appropriate tests (e.g. normality and sphericity for ANOVAs; normality of residuals and outliers/high influence observations for mixed effects models) and visual inspection. Non-normality was addressed by either evaluating results with a non-parametric version of the statistical test under question (e.g. Wilcoxon Signed Rank test instead of *t*-test) or by using robust standard errors (in mixed modeling analyses). Modeling analyses were run with and without observations yielding large residuals and/or having high influence. In all cases where these robustness checks were performed, the conclusions for the primary effects of interest were the same.

#### Provider outcomes.

To evaluate achievement of competency in CBT-I, the number of therapists demonstrating competency on the CBT-I CRS, as reflected by a total CBT-I CRS score of ≥30 and ratings ≥2 on five core competency items, at the end of the training consultation phase was calculated. CBT-I CRS data were available for all 11 therapists.

Changes in therapist knowledge, attitudes, and self-efficacy to deliver CBT-I were evaluated using a 3 (domain) × 3 (time) within-participants ANOVA. The levels for the two factors were Knowledge, Attitudes, and Self-Efficacy (domain), and Pre-Workshop, Post-Workshop, and Post-Consultation (time). Post hoc tests consisted of one-way (time) within-participants ANOVAs for each rating domain, followed by paired-sample *t*-tests for consecutive time points. Hedge’s *g,* which corrects for small sample sizes, was reported to estimate the magnitude of differences across time points [55]. Data for the therapists that left training prior to completion were available for Pre- and Post-Workshop but not at Post-Consultation. Inclusion of her data did not impact the results of any analyses of therapist ratings. To simplify the interpretation of the scores across comparisons, her data were removed from all analyses of knowledge, attitudes, and self-efficacy.

#### Patient outcomes.

Patient outcomes on the ISI were first analyzed descriptively. Changes in both ISI score and percent change in ISI were calculated. Changes in ISI scores were categorized based on achieving moderate to marked improvement (change in score of ≥8) [53]. Given that the PHQ-9 was systematically administered only at intake and final session, reflecting the real-world effectiveness nature of the evaluation, descriptive statistics were calculated using data from those who completed treatment. For the PHQ-9, the percentage of patients meeting each severity classification at the intake and final sessions were calculated, as well as the percent change in PHQ-9 score from intake to final session. For both the ISI and PHQ-9, effect sizes for the mean change (Hedge’s *g*) are also provided.

Changes in patient scores on the ISI were also analyzed inferentially using linear mixed-effects regression. The ISI scores immediately prior to starting CBT-I (session 1) and at the start of the last session were used in mixed effects models. Models with ISI scores as the outcome are ITT unless otherwise stated (including all patients regardless of whether they completed treatment). Fixed effects predictors included in the model were patients’ age (standardized), sex (male or female), race (categorical; white/Caucasian or black, other, did not disclose), insurance type (categorical; commercial, Medicare Advantage, Medicaid, or other), number of sessions completed, and time (categorical; second, or final). For the latter, “second” refers to the second appointment, which was the first treatment session following intake. Race was entered as a binary variable due to low frequencies of patients identifying as races other than white or Caucasian. A random intercept by the patient was included to control for the correlation in scores across sessions. The primary predictor of interest was time—the estimated change in the modeled outcome from the beginning of treatment to the final session attended by the patient.

Additional models that were otherwise identical to those described above were conducted to clarify the magnitude of changes in ISI scores. For the first of these models, an interaction between time and number of sessions completed was included to estimate the incremental effect of each CBT-I session (i.e. a dose effect). For the second model, the data included were limited to patients who completed treatment to provide an estimate of the maximum anticipated treatment effect. In a final set of models, the sample was split into two groups to examine CBT-I outcomes between patients with (*n* = 11) and without (*n* = 37) any active insomnia-related prescription medication. In this model, the primary ITT model described above was re-run within these two subgroups to explore whether medication use impacted outcomes.

## Results

### Provider outcomes

Over the course of the 5-month training consultation process, all 11 therapists reached competency (i.e. CBT-I CRS score ≥30 and ratings ≥2 on five core competency items) and successfully completed all program requirements. Therapists’ ratings of knowledge, attitudes, and treatment self-efficacy changed over time, but did so to different degrees across domains. These differential changes were indicated by a significant domain by time interaction, *F*(1.81, 18.09) = 24.90, *p* <. 001. Follow-up tests revealed significant simple main effects of time within the knowledge (*F*[2, 20] = 42.09, *p* <.001) and self-efficacy domains (*F*[2, 20] = 29.44, *p* <.001) but not Attitudes (*p* =.42). Mean ratings across items in each domain at pre- and post-workshop and post-consultation are shown in the top of Table 1. Mean scores for items reflecting knowledge of insomnia and CBT-I (knowledge) increased significantly from pre- to post-workshop, *t*(10) = 7.00, *p* = <.001 (Hedge’s *g* = 2.94) but not from post-workshop to post-consultation, *t*(10) = 1.12, *p* = .25 (*g* = 0.53). Mean scores for items reflecting confidence in assessing insomnia and implementing CBT-I (Self-Efficacy) increased significantly from pre- to post-workshop, *t*(10) = 4.05, *p* = 0.005 (*g* = 1.82) and from post-workshop to post-consultation *t*(10) = 4.27, *p* = 0.003 (*g* = 2.02).

**Table 1.** Average Total Score for Each Domain of Therapist Self-ratings and Averages for Individual Items Eliciting Therapists’ Evaluation of the Training Program

Assessment or domain/item	Pre-workshop *M* (*SD*)	Post-workshop *M* (*SD*)	Post-consultation *M* (*SD*)
*Therapist self-ratings*			
Knowledge	21.64 (4.23)	33.91 (3.45)	35.82 (3.22)
Attitudes	26.73 (1.62)	27.09 (1.30)	27.36 (1.03)
Self-Efficacy	27.18 (10.53)	42.18 (3.89)	50.09 (3.33)
*Therapists’ evaluation of program*			
Overall satisfaction with CBT-I training ^a^	—	—	6.64 (0.49)
Training increased overall job satisfaction ^b^	—	—	5.64 (0.78)
Training improved skills in CBT-I ^b^	—	—	7.00 (0.00)
Training improved overall skills as a therapist ^b^	—	—	6.18 (0.85)
Plan to use CBT-I as a result of training ^c^	—	—	6.73 (0.63)

*N* = 11. The minimum and maximum possible domain scores for self-ratings were 6 and 42 for Knowledge, 4 and 28 for Attitudes, and 8 and 56 for Self-Efficacy. All individual items for self-ratings and program evaluation had a minimum and maximum possible score of 1 and 7, respectively. ^a^ 1 = Not at all satisfied, 7 = Extremely satisfied; ^b^ 1 = Not at all, 7 = A great deal; ^c^ 1 = Strongly disagree, 7 = Strongly agree.

Therapists’ ratings of their experience and satisfaction with the training are displayed in Table 1. As the results reveal, ratings were high across all areas (maximum score of 7), as evidenced by scores of 5 or higher across all items (mid-point of scale = 4, meaning “Somewhat” or “Somewhat Satisfied/Helpful”). All therapists indicated the training improved their skills in CBT-I “A Great Deal” (*M* = 7 out of 7) and that the training also improved their *overall* skills as a therapist (*M* = 6.2, *range* = 5 to 7). Therapists were highly satisfied with the program (*M* = 6.6, *range* = 6 to 7), and indicated the training also improved their overall job satisfaction (*M* = 5.6, *range* = 5 to 7). Importantly, therapists indicated high intention to use CBT-I as a result of training (*M* = 6, *range* = 5 to 7).

### Patient outcomes

A total of 48 patients attended at least one treatment session (session 1) following initial intake. Descriptive statistics for demographics, behavioral and physical health status indicators, and treatment completion status are provided in Table 2. In brief, the average patient age was 53.8 (*range =* 22 to 88), a slight majority of patients were female, and nearly all identified as white. Overall, patients had few major physical health comorbidities, as reflected by the low mean Charlson Comorbidity Index score (sample mean < 1). Comorbid anxiety disorders were common in the sample, occurring in nearly half of patients. More than 1 in 4 patients had a cooccurring, non-insomnia sleep disorder and about 1 in 5 had an active sleep medication. In terms of treatment completion status, more than two-thirds of the patient sample (68.8%) completed treatment (completed all sessions in the treatment protocol or finished early due to symptom relief), and more than 85% of patients completed at least two treatment sessions in addition to the initial intake session. Additional details on treatment completion status are displayed in Table 2.

**Table 2.** Patient Characteristics and Treatment Completion Status Among Those Who Completed at Least One Treatment Session

Characteristic	Mean (SD)/ *n* (%)
Age	53.8 (16.9)
*Sex*	
Female	33 (68.8%)
Male	15 (31.2%)
*Race*	
White	46 (95.8%)
Other	1 (2.1%)
Patient declined	1 (2.1%)
*Insurance type*	
Private	31 (64.6%)
Medicare	11 (22.9%)
Medicaid	3 (6.3%)
Other	3 (6.3%)
Non-insomnia sleep disorder	13 (27.1%)
Prescription for sleep aid	11 (22.9%)
Charlson Comorbidity Index score	0.3 (0.7)
Depression	15 (31.2%)
Anxiety	23 (47.9%)
PTSD	3 (6.2%)
Alcohol use disorder	1 (2.1%)
Other substance use disorder	0 (0%)
*Treatment completion status*	
Completed ≥3 sessions	41 (85.4%)
Completed treatment	33 (68.8%)
Completed in <6 sessions due to symptom relief	11 (33.3%)
Discontinued treatment	11 (22.9%)
Lost to follow-up due to unknown reasons	4 (8.3%)

*N* = 48 patients completed ≥ 1 treatment session. Treatment completion was defined collaboratively by therapist and patient.

Across all patients, the average number of completed sessions was 5.8 (*SD* = 2.6, *range* = 2 to 13 sessions), and the median number of completed sessions was 5.5. A large percentage of patients who attended at least one treatment session achieved at least moderate to marked reduction in insomnia severity (decrease of ≥ 8; 45.8%, *n* = 22). Among those who completed treatment (*n* = 33), 60.6% (*n* = 20) achieved at least a moderate to marked reduction in insomnia severity.

**Figure 1.**
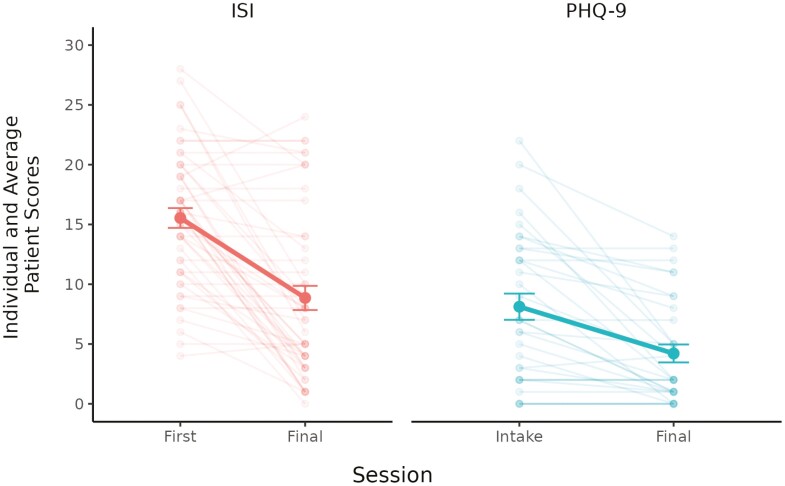
Improvement in average (bold line) and individual scores from immediately prior to treatment (session 1) to after (final session). Error bars represent +/− 1 standard error.

The visual decrease in ISI scores in Figure 1 was confirmed by the ITT analyses, with mixed effects models (Table 3) showing that time (first treatment session vs. final session) was a significant predictor of ISI scores (b = −6.69, 95% CI: −8.59 to −4.79, *p* < .001). Averaging over patient characteristics, the average ISI score at the start of treatment was 12.57 (*SE* = 1.08) and reduced to 5.88 (*SE* = 1.14). The ITT effect based on the raw means was large (*g* = 0.99). The additional models revealed significant sessions completed by time interaction (b = -1.57, 95% CI: −1.96 to −1.17, *p* < .001), indicating that reductions in ISI scores grew with more CBT-I sessions completed. In the model limited to patients who completed CBT-I (*n* = 33), time significantly predicted reduced ISI scores (b = −9.12, 95% CI: −11.11 to −7.13, *p* < .001). Averaging over patient characteristics, those who completed CBT-I had an average ISI score of 12.85 (*SE* = 0.77) at the start of treatment, which reduced to an average of 3.73 (*SE* = 0.87), an average improvement of 71%. The effect among those completing treatment was large, and higher than that in the ITT sample (*g* = 1.56). Finally, the models examining change in insomnia severity among those with and without an active sleep medication revealed that patients without an active sleep medication showed large reductions in insomnia severity (b = −7.03, *SE* = 1.06, *p* < .001, *g* = 1.06); and those with an active sleep medication showed moderate to large reductions in insomnia severity (b = −5.55, *SE* = 2.21, *p* = .026, *g* = 0.70).

**Table 3.** Results of the Linear Mixed Effects Models Evaluating Overall Changes in ISI Scores Among the Full Sample (ITT), Incremental Change as a Function of Sessions Completed (Dose Effect), and Among Those Who Completed Treatment (Completed Only)

	ISI—ITT			ISI—Dose Effect			ISI—Completed Only		
Predictors	*Estimates*	*95% CI*	*P*	*Estimates*	*95% CI*	*P*	Estimates	*95% CI*	*P*
(Intercept)	18.93	14.50 to 23.36	**<.001**	14.41	9.84 to 18.98	**<.001**	9.62	5.98 to 13.25	**<.001**
Age (standardized)	0.56	−1.51 to 2.63	.592	0.56	−1.51 to 2.63	.592	0.53	−0.59 to 1.65	.349
*Sex*									
Female									
Male	−1.23	−5.66 to 3.20	.582	−1.23	−5.66 to 3.20	.582	0.03	−2.48 to 2.54	.981
*Race*									
White									
Black, other, declined	−4.69	−7.76 to −1.62	**.003**	−4.69	−7.76 to −1.62	**.003**	−4.3	−6.60 to −2.00	**<.001**
*Insurance type*									
Private									
Medicare	−3.17	−8.82 to 2.48	.268	−3.17	−8.82 to 2.48	.268	−3.43	−6.39 to −0.47	**.024**
Medicaid	3.7	−0.18 to 7.58	.062	3.7	−0.18 to 7.58	.062	3.73	1.30 to 6.15	**.003**
Other	−6.52	−14.11 to 1.07	.091	−6.52	−14.11 to 1.07	.091	−2.36	−6.67 to 1.95	.277
Sessions completed	−0.33	−1.01 to – 0.35	.336	0.45	−0.21 to 1.12	.181	0.86	0.31 to 1.40	**.003**
*Time*									
Second									
Final	−6.69	−8.59 to −4.79	**<.001**	2.35	−0.34 to 5.05	.086	−9.12	−11.11 to −7.13	**<.001**
Sessions completed × time [final]	—	—	—	−1.57	−1.96 to −1.17	**<.001**	—	—	—
*Random effects*									
σ^2^	21.94			13.65			16.21		
τ_00_ _PatientID_	16.98			21.12			3.95		
ICC	0.44			0.61			0.2		
N _PatientID_	48			48			33		
Observations	96			96			66		
Marginal R^2^/conditional R^2^	0.310/0.611			0.385/0.758			0.585/0.667		

*CI,* Confidence Interval.

As with ISI scores, PHQ-9 scores decreased from intake to the final assessment. The right panel of Figure 1 shows the average and individual changes from baseline to posttreatment scores. Among those who completed CBT-I (*n* = 33), the percentage of patients experiencing moderate or greater depression severity (PHQ-9 ≥ 10) at baseline was 42.4% (*n* = 14), with median score of 7 (*M* = 8.21, *SD* = 6.30, *range* = 0 to 22). At posttreatment, 15.2% (*n* = 5) of patients were experiencing moderate depression (PHQ-9 of 10 to 14), and no patients were experiencing moderate-severe or severe depression. Posttreatment PHQ-9 median score was 2 (*M* = 4.21, *SD* = 4.31, *range* = 0 to 14). The decrease in PHQ-9 scores from intake to the final session was large (*g* = .81, 95% CI: 0.62, 1.08).

## Discussion

Insomnia is associated with substantial clinical and financial costs and considerable unmet need. In spite of its high treatability and wide-reaching impact across physical, psychological, social, economic, well-being, and quality of life domains, insomnia is highly under-treated. The current article examined initial outcomes associated with the real-world implementation of gold-standard insomnia treatment in routine primary care and specialty behavioral health settings as part of a pilot initiative designed to assess feasibility and effectiveness.

The high prevalence and considerable costs associated with insomnia [24] served as a key basis for the pilot implementation initiative to establish new capabilities in CBT-I within routine primary care and behavioral health settings within AHN. Initial outcomes from CBT-I implementation demonstrate considerable effectiveness of training in and delivery of CBT-I in these settings, as evidenced by robust improvements at both provider and patient levels. Among providers, training in CBT-I was associated with large increases in preparedness to deliver the intervention. Notably, all clinicians (excluding 1 clinician who changed employment early in the training process) successfully completed the training program, including process-oriented and competency-based completion criteria. In addition, provider outcome data reveal large increases in knowledge and, especially, confidence to deliver CBT-I. Moreover, it is noteworthy that, beyond improvements in CBT-I knowledge, skills, and confidence, the training process increased *overall* therapy skills and job satisfaction. These findings are consistent with provider-related outcomes associated with the implementation of CBT-I within the VA health care system; within VA, 93% of providers participating in CBT-I training successfully achieved competency and completed all training program requirements [45]. The findings are also consistent with findings of positive changes in knowledge, skills, and treatment self-efficacy reported in similar CBT competency-based training initiatives within the VA and Kaiser Permanente systems [50, 51].

Among the most promising findings of the study is that the experience of receiving CBT-I from busy, newly trained clinicians was associated with large improvements in insomnia severity, as well as improvements in comorbid depression severity. Close to half of patients receiving at least one treatment session (beyond an intake session), and nearly two-thirds of those completing the brief treatment, achieved clinically significant [53] reductions in insomnia severity. Furthermore, the average level of symptom reduction among those completing this brief treatment was 71%.

The clinical outcomes observed in the current evaluation are comparable to results reported in RCTs of CBT-I [56, 57] and in the implementation of CBT-I within the VA health care system. The mean reduction in the ISI of 53% (based on ITT analysis) in the current evaluation is in line with overall ISI improvement rates of 48% in a seminal CBT-I RCT and 47%–49% in VA [44–46]. The foregoing notwithstanding, it is important to note that, while in the clinically significant range, the mean level of insomnia severity among patients receiving CBT-I at baseline in the current initiative was lower than that among Veterans presenting for treatment in the VA health care system, as well as participants in other studies; further, patients in the current study, overall, had relatively few major physical health comorbidities. The current findings provide valuable insight suggesting a positive response to and utility of CBT-I among those with less severe clinical profiles. Additional research would help to elucidate whether the effects documented herein may extend to private system patients with more severe and complex presentations. Additional implementation experience within AHN will allow for further evaluation of this.

Of note, the rate of treatment completion (completed all sessions or finished early due to symptom relief) in the current study (69%) is very similar to the corresponding rate (67%) in the implementation of CBT-I in the VA health care system [45]. While there is an opportunity to increase ongoing engagement in CBT-I and other psychotherapies, it is important to note that in both initiatives, patients were undergoing treatment by clinicians new to the intervention and in the process of undergoing training in the treatment. In addition, the fact that patients generally did not have severe or complex presentations in the current evaluation may further support the treatment completion rate not being higher as perceived need in less complex or severe cases is typically reduced. In fact, increasing research has shown that engagement in and intensity of mental health treatment utilization is associated with higher levels of perceived severity, a phenomenon that is likely mediated by distress and impairment [58, 59].

Beyond differences in patient mix and presentation, there were several additional factors distinguishing the CBT-I implementation experience in the current initiative from the implementation of CBT-I and other EBPs in the VA system. First, the VA health care system is a closed system (i.e. has full control over its providers and payment of services), whereas Highmark Health, as a payor-provider organization, operates within an open system and networks of providers that are reimbursed by various insurance plans and other payors. As a closed system, VA instituted national policy and top-down mechanisms to promote implementation and sustainability, including system requirements for CBT-I availability at each medical center. Moreover, while financial considerations are important in shaping services in all health care delivery systems, issues of cost-effectiveness are particularly salient in private and payor systems. Accordingly, it was important in the current initiative to demonstrate significant clinical and financial need and opportunity associated with untreated and undertreated insomnia to support the current implementation effort. In so doing, we found that median total health care costs among members with insomnia were 4–6 times greater than among those without insomnia, and mean total costs were 2–3 times greater [24]. There are plans for actively monitoring through claims data analysis the health care utilization and costs for members who received CBT-I, a process much more readily feasible within a payor-provider system. Positive findings related to cost-effectiveness could further enhance efforts to sustain and scale CBT-I.

In addition, AHN, like many private (and public) systems with limited experience with formal insomnia treatment implementation, had a highly medical (pharmacological) orientation and culture for treating insomnia prior to and during the current implementation initiative. This was unique from the treatment culture within primary care within VA, though this was an implementation barrier early on in the implementation experience in the VA. Like many systems, patients with insomnia frequently presented to primary care with sleep-related complaints which, if treated, were commonly managed with hypnotics or other sleep-related medications. Further, there was limited awareness of and confidence in CBT-I for addressing the needs and demands of patients presenting for treatment for insomnia, which initially contributed to lower-than-expected referrals for CBT-I. Accordingly, careful attention was placed on establishing processes and procedures for identifying insomnia and referring appropriate patients to trained providers.

Furthermore, organized processes were established for the CBT-I-trained providers to meet with primary care and other staff to promote knowledge of confidence in CBT-I and serve as local implementation champions of the intervention. This bottom-up mechanism of implementation support became a key area of focus and important facilitator of implementation within VA that included the placement of designated local champions of EBP implementation, known as Local EBP Coordinators [44]. While important, top-down policy requirements are not sufficient to promote and sustain EBP delivery.

Over time, primary care and other staff at AHN witnessed the impact of CBT-I and came to appreciate its place within the clinical infrastructure, bringing changes to the treatment culture and professional demand for the services. In fact, the experience led to significant interest among primary care providers in establishing CBT-I treatment capacity within primary care practices that did not have a trained provider, as well as support from clinical leadership. In addition, the experience led to significant demand for training among behavioral health providers who were not part of the initial training cohort. Plans are currently underway for training additional staff and increasing CBT-I treatment capacity.

One additional note related to the barriers and facilitators of CBT-I implementation in the current initiative concerns the lack of 50–60-minute treatment sessions for delivering CBT-I within primary care and the fact that the Collaborative Care Model that was in place in the primary care implementation sites is typically brief in nature. To enable implementation, adaptations were made to the training and treatment to allow for delivery of CBT-I within 30-minute sessions (with the exception of the longer initial evaluation session) within primary care clinics. This adjustment, important for both initial implementation and sustainability and scale, was successfully implemented. This and the other clinical and systems mechanisms described above for promoting implementation and sustainability will, with more time and maturation, likely have an even greater impact in future phases of implementation.

While the current results represent initial findings from the implementation of CBT-I in a real-world, private system setting, the significant effectiveness of CBT-I observed in this context further speaks to the importance of—and opportunity for—treating insomnia and for considering insomnia for initial focus in individuals with cooccurring insomnia and other behavioral health conditions [60]. Notably, because of its brevity and highly behavioral focus (as opposed to a focus on emotional domains or unresolved childhood or family dynamics), some individuals are often more willing to initiate CBT-I than other behavioral health treatments. Although many individuals demonstrate significant improvements in comorbid symptoms (further supported by the current evaluation) who do not necessitate further treatment, successful experience in CBT-I can increase motivation and engagement in those who may benefit from additional psychological treatment. As such, CBT-I presents a significant opportunity as a gateway intervention.

Beyond its clinical impact on insomnia (and cooccurring behavioral health disorders), CBT-I has recently been shown to have notable preventive effects on depression, leading to calls for it to be considered as a key public health approach to addressing depression [61, 62]. For example, Irwin and colleagues found that CBT-I decreased the likelihood of depression by over 50% as compared to sleep education therapy in adults over the age of 60 with insomnia [63]. Indeed, patients receiving CBT-I in the current study, which was associated with robust improvement in depression severity similar to past studies [45, 47], had a median PHQ-9 score of 2 (no depression) at the end of this brief treatment; and the number of patients having at least moderate depression reduced by almost two-thirds within this short time-frame.

It is important to consider additional limitations of the current study when interpreting the current findings. As a real-world effectiveness evaluation, the examination of the impact of CBT-I on insomnia severity did not include a control group. At the same time, support for the current results is enhanced by the finding of a “dose effect,” or incremental improvements with additional sessions completed. Additional support for the observed effects being attributable to CBT-I as opposed to the passage of time or other factors is the large treatment effect observed, which is in line with controlled research on and effectiveness evaluations of CBT-I and the fact that comparison groups in these studies typically show limited improvement in insomnia, which is often enduring in nature [56, 57, 64].

Furthermore, while data on cooccurring physical and behavioral health conditions and medications that could impact insomnia outcomes were included, the present sample was not large enough to conduct formal moderation analyses with these variables. In exploratory models, the effects of CBT-I were independent of prescription sleep medication use such that those without a prescription demonstrated large reductions in insomnia severity and those with an active sleep medication received comparable benefits from CBT-I. However, given the small sample sizes, the results of these analyses should be considered preliminary and require replication. Furthermore, while the sleep medications were active prescriptions, information regarding medication adherence and consumption patterns was not available. An additional limitation of the current study is the lack of data on the use of over-the-counter medications.

It is also important to note that patients receiving CBT-I as part of this initial implementation were predominantly white/Caucasian and included individuals with insurance. As a result, the generalizability of the results should be considered when applying the findings to individuals dissimilar to (and more heterogenous than) the sample in regard to insurance status, ethnicity, clinical status, and other factors.

It is also worth noting the potential for findings to have been influenced by the specific make-up of the cohort of clinicians (and patients) who applied to and participated in the program. While clinicians were not required to have received any formal EBP training or to meet a certain threshold for clinical experience or expertise, clinicians were considered based, in part, on their interest in training and at least general interest in CBT, more broadly. Indeed, pre-workshop survey results reveal overall favorable attitudes of the training cohort (though significantly lower ratings of knowledge and self-efficacy) toward CBT-I at the outset of training. This process was similar to the corresponding process employed in the EBP dissemination and implementation initiatives in the VA health care system and other systems, where clinician motivation was generally high, especially at the outset of the implementation process [50]. In fact, in these and the current initiatives, efforts were undertaken to promote motivation by framing the initiatives as part of a transformation process and through the sharing of outcome data and patient success stories from similar initiatives prior to the start of the training process. Nevertheless, in our past work over years of implementation of CBT-I and other EBPs in VA and other systems, we found generally consistent results across a large number of training cohorts [44–47, 50]. Intentionally, this work, as in the current implementation initiative, began with a focus on motivated clinicians who could serve as early adopters; over time, the positive results and experiences of these earlier cohorts helped to increase interest and motivation among those initially less motivated (later adopters). Future CBT-I training and implementation efforts should account for the level of provider motivation in recruitment and overall implementation planning and should include examination of the effects of such on outcomes. To the extent that CBT-I is delivered in systems and clinics where clinician interest may be low, settings may be less appropriate for psychological treatment of insomnia, and there is little overall focus on implementation climate and readiness, there is a possibility for outcomes to be attenuated.

Furthermore, it is important to highlight that competency ratings and clinician ratings of knowledge, attitudes, and treatment self-efficacy were collected during and immediately following the training process. As such, there is potential for specific skills to erode over time, especially if not utilized, and for therapist adoption to drift following intensive training and ongoing consultation. Accordingly, to help promote sustained skills and adoption, ongoing informal peer consultation was established and recommended as a structure and mechanism by which the trained clinicians continue to meet without the formal training consultants, a process incorporated into other EBP training and implementation initiatives. In fact, in our experience, we have observed many consultation groups voluntarily pursue this on their own as they come to highly value the consultation process. In addition, designated local champions can help to facilitate ongoing skill mastery and sustainability. This is a specific duty of the Local EBP Coordinators in the VA system. Similar informal champion support is a role beginning to be informally developed within AHN.

Lastly, because the PHQ-9 was not systematically re-administered until the end of CBT-I treatment, reflecting the real-world effectiveness nature of this evaluation and considerations of patient impacts, ITT analysis of the impact of CBT-I on depression symptoms was not possible; nevertheless, the large reduction in depression severity provides support for the broader potential benefit this treatment can provide, consistent with past controlled studies and effectiveness research [45, 64, 65].

In sum, the foregoing results related to the treatability of insomnia suggest that greater attention be paid to addressing insomnia. The current findings, which represent initial outcomes from the structured implementation of CBT-I in routine treatment settings within a private health system context, add to and extend findings regarding the feasibility and effectiveness of training in and implementation of CBT-I within the VA health care system, as well as recent findings related to real-world training in and implementation of evidence-based psychological treatment focused on depression and other behavioral health conditions within Kaiser Permanente and other systems [40, 45, 47, 50, 66]. Collectively, the provider and patient-level outcomes and experiences reported herein point to the potential benefits, at multiple levels, that increasing the availability of gold-standard treatment for insomnia may confer to private payor and provider health systems. Additional research on the effects of CBT-I implementation, incorporating larger samples of providers and patients, within private systems and on moderators of patient, provider, and system-level outcomes and sustainability is encouraged.

## Data Availability

The data underlying this article cannot be shared publicly due the use of private health information and proprietary data.
